# The rise of neglected tropical diseases in the "new Texas"

**DOI:** 10.1371/journal.pntd.0005581

**Published:** 2018-01-18

**Authors:** Peter J. Hotez

**Affiliations:** 1 Texas Children’s Hospital Center for Vaccine Development, Departments of Pediatrics and Molecular Virology and Microbiology, National School of Tropical Medicine, Baylor College of Medicine, Houston, Texas, United States of America; 2 Department of Biology, Baylor University, Waco, Texas, United States of America; 3 James A Baker III Institute for Public Policy, Rice University, Houston, Texas, United States of America; 4 Scowcroft Institute of International Affairs, Bush School of Government and Public Service, Texas A&M University, College Station, Texas, United States of America; FIND, SWITZERLAND

## Abstract

Within the last five years, the State of Texas has experienced either transmission or outbreaks of Ebola, chikungunya, West Nile, and Zika virus infections. Autochthonous transmission of neglected parasitic and bacterial diseases has also become increasingly reported. The rise of such emerging and neglected tropical diseases (NTDs) has not occurred by accident but instead reflects rapidly evolving changes and shifts in a “new” Texas beset by modern and globalizing forces that include rapid expansions in population together with urbanization and human migrations, altered transportation patterns, climate change, steeply declining vaccination rates, and a new paradigm of poverty known as “blue marble health.” Summarized here are the major NTDs now affecting Texas. In addition to the vector-borne viral diseases highlighted above, there also is a high level of parasitic infections, including Chagas disease, trichomoniasis, and possibly leishmaniasis and toxocariasis, as well as typhus-group rickettsiosis, a vector-borne bacterial infection. I also highlight some of the key shifts in emerging and neglected disease patterns, partly due to an altered and evolving economic and ecological landscape in the new Texas, and provide some preliminary disease burden estimates for the major prevalent and incident NTDs.

## Introduction: The new Texas

Texas has become one of the fastest growing US states, with projections that by 2020, the population will exceed 30 million people [1]. A majority are expected to live in metropolitan urban areas [1]. Population growth and urbanization are accelerating at an impressive rate. According to United States Census data released in 2016, Texas now hosts five of the 11 fastest growing small cities and five of the eight fastest growing large cities (Houston, San Antonio, Fort Worth, Dallas, and Austin) [2]. Growth is due to immigration from both US northern states and abroad, mostly for economic opportunities. In 2014, it was estimated that more than 4.5 million Texans are foreign born, including more than 3 million from Latin America and almost 1 million and 200,000 from Asia and Africa, respectively [3]. Texas is ranked third among US states in percentage of foreign-born population [4].

Into this mix of rapid growth, urbanization, and immigration are additional and critical factors that could promote the emergence of infectious and neglected diseases [5–8]. First is the rise in poverty in Texas. Despite the state’s enormous wealth and an overall economy that exceeds Canada, Australia, or Russia [9], Texas also has profound pockets of intense poverty. Poverty has been shown to be an overwhelming social determinant for the promotion of neglected tropical diseases (NTDs), and today, most of the poverty-related NTDs and emerging infections are found in concentrated areas of poverty hidden in wealthy economies—a concept known as “blue marble health” [10]. Texas ranks in the bottom tier of US states in terms of high poverty levels (37th in terms of overall poverty and child poverty) [7], but because of its large overall population, with more than 4 million Texans living below the poverty line in 2016, Texas also ranks among the states with the largest number of people living in poverty of any US state and near the bottom (41st) in terms of food insecurity and educational attainment [11].

**Fig 1:**
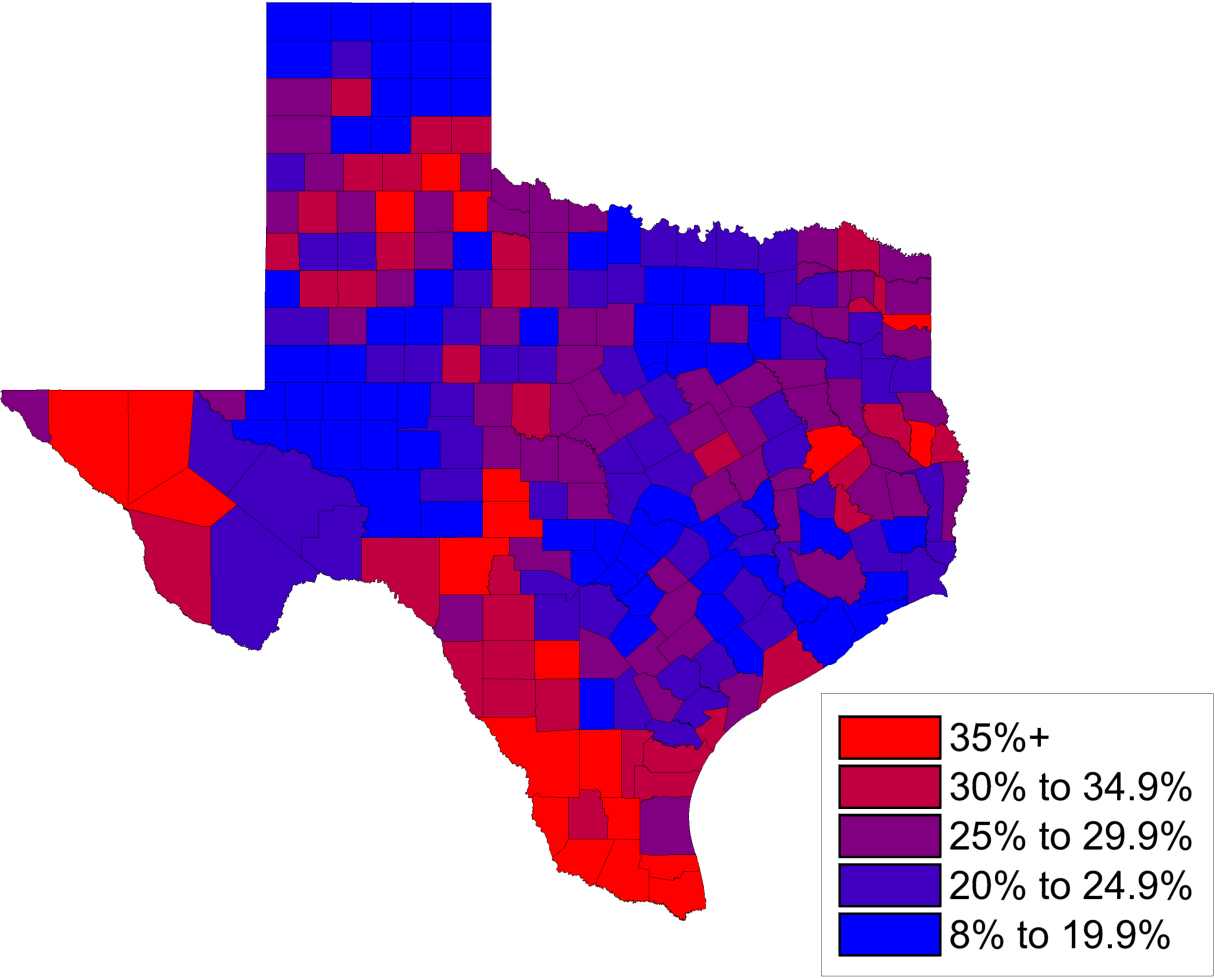
Percentage of children in poverty ages 0 to 17 by county in Texas in 2015. Original map drawn by Dr. Melissa Nolan and Mr. Nathaniel Wolf based on data from the US Census Bureau, accessed at http://www.census.gov/did/www/saipe/data/statecounty/data/2015.html.

**Fig 2:**
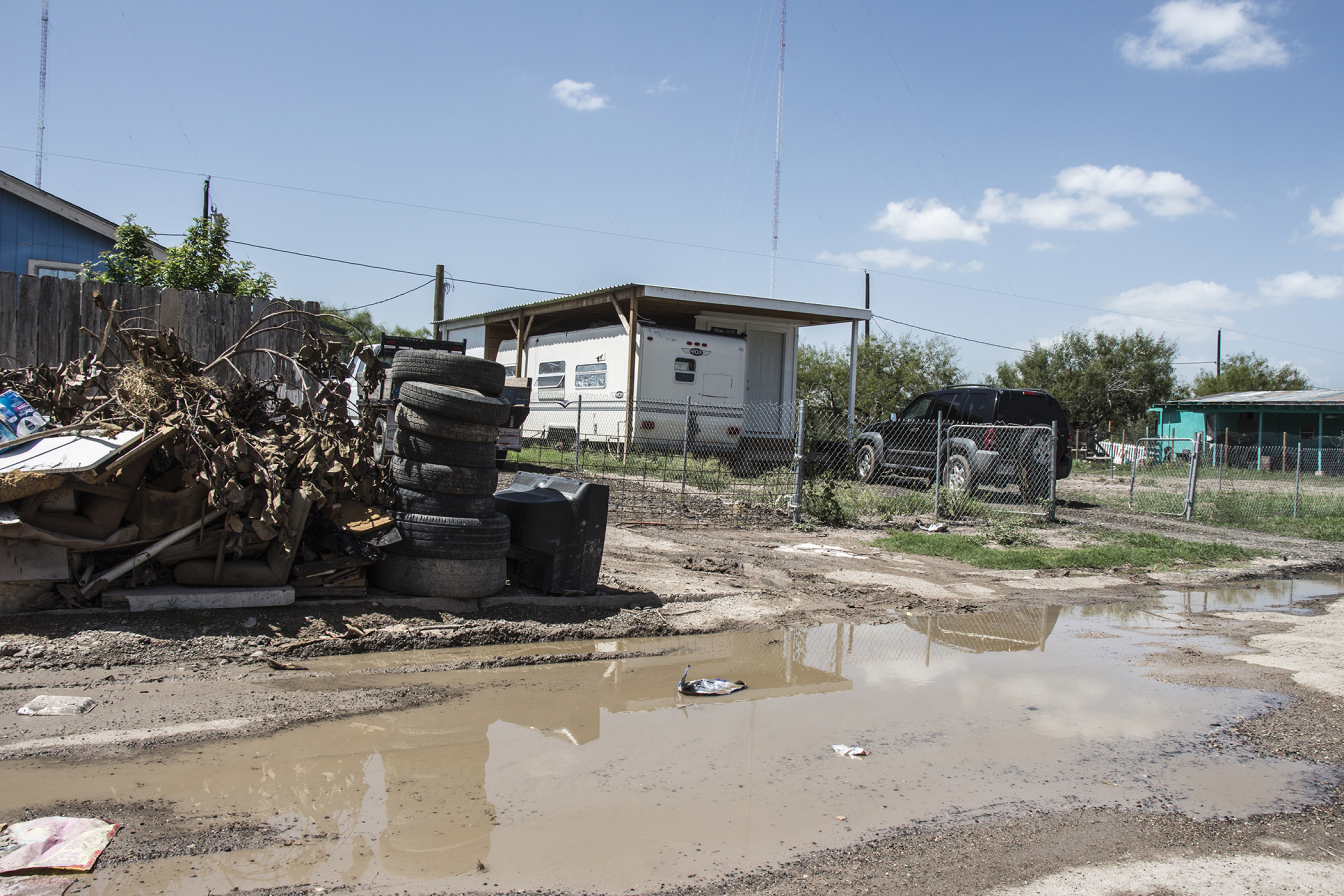
Colonia Mi Sueño, Rio Grande Valley, Texas. Photo by Shaghayegh Tajvidi.

Fig 1 shows a map of child poverty in Texas indicating that poverty is not evenly distributed but instead is focused in South Texas and along the border with Mexico, in addition to focal areas of East Texas and the Texas Panhandle. Especially noteworthy are the so-called “colonias”—unincorporated residential communities near the Mexico border that often lack basic services, including access to clean water, sewer systems, and paved roads (Fig 2) [12]. The major urban areas and cities of Texas also experience high levels of poverty, with child poverty rates exceeding 33% in Dallas and Houston [13].

In addition to poverty, trade and human migrations also contribute to the risk of NTDs in the state of Texas. With regards to the former, trade is growing massively due to a doubling in size of the Panama Canal and expansion in size for more than a dozen Texas ports, including Port Arthur, Houston, Corpus Christi, and Brownsville, among others, which generate close to $100 billion in annual revenue [14] but also expose Texas populations to shipping traffic and potential diseases from Asia, Europe, and Africa [8]. Similarly, there is massive traffic across the Rio Grande River from Mexico that facilitates human migrations as well as invasions of exotic plant species that promote tick and other vector survival [15].

There is also the threat of climate change. Among US states, Texas is disproportionately at risk to global warming and by some estimates, Texas is projected to reach 80–100 days over 95°F by the 2050s, compared to approximately 40 such days over the last 30 years) [16]. The state will face rising sea levels and other negative effects.

Of particular relevance and concern are the warming effects conductive to insect vector expansion and transmission, which could alter current mosquito, sandfly, flea, and kissing bug distributions [15]. Finally, it has been noted that a powerful anti-vaxxer movement and lobby is causing 50,000 or more parents to file nonmedical exemptions for childhood and school entry vaccines so that Texas is now at risk for the emergence of vaccine-preventable diseases, especially measles [17]. The factors of rapid population expansion, human migrations, urbanization, poverty, climate change, declining vaccine rates, transborder traffic, and sea transportation shifts have combined to create a new Texas that is particularly suited for the rise of emerging and neglected diseases (Box 1).

**Box 1. Major external factors driving emerging and neglected diseases in Texas**

- Population expansion
- Human migrations
- Urbanization
- Poverty
- Climate change
- Declining vaccinations
- Transborder traffic
- Sea transportation

## Methods

Here I review the current status of NTDs in Texas using a search conducted in December 2016 emphasizing new information published within the previous five years. I used the online database PubMed from 2012 to 2016 with the Medical Subject Headings, the specific diseases listed as NTDs on the *PLOS Neglected Tropical Diseases* website (http://www.plosntds.org/static/scope.action, in addition to several selected diseases that have been reviewed regularly at *PLOS NTDs*, including trichomoniasis, cryptosporidiosis, and borreliosis), and the geographic region of Texas. Reference lists of identified articles and reviews were also hand searched as were databases from the Texas Department of State Health Services (DSHS; https://www.dshs.texas.gov/). In a few instances, articles prior to 2012 were also cited when relevant, while some of the findings were also updated when relevant a year later in December 2017.

## Findings

Major findings were related to the following three major groups of NTDs: neglected parasitic infections, arthropod-borne virus (arbovirus) infections, and other neglected viral and bacterial infections.

### Neglected parasitic infections

The major neglected parasitic infections in Texas include Chagas disease, leishmaniasis, trichomoniasis, intestinal protozoan infections, cysticercosis, and toxocariasis. A unique feature about Chagas disease (American trypanosomiasis caused by *Trypanosoma cruzi*) is that Texas represents one of the few US states in which autochthonous transmission occurs or may be common. Triatomine kissing bug vectors were first described in South Texas during the 1930s, with local human Chagas disease cases associated with triatomines reported in the 1940s and 1950s [18]. In 2015, five patients with autochthonous Chagas disease from southeast Texas were identified through blood screening [19], with counties in South Texas considered especially high-risk areas [15, 20]. A subsequent study looking at risk factors for *T*. *cruzi*–positive blood donors included poverty, with older and minority persons at greatest risk [21]. Cardiac manifestations and evidence of Chagasic cardiomyopathy have also been noted among these populations [22]. Sylvatic transmission has also been proposed, including Chagas disease cases among hunters and campers [23, 24]. Human Chagas disease is seldom diagnosed in Texas and is consequently underreported. Between 2013 and 2015, there were only 15 locally acquired cases and 44 imported cases reported to DSHS [25]. The Centers for Disease Control and Prevention (CDC) estimates that there are almost 37,000 people living with Chagas disease in Texas [26], although the percentage of cases transmitted locally versus through immigration across the southern border remains unknown. *T*. *cruzi* meningoencephalitis in patients with HIV/AIDS has also been described in Texas [27].

The complete ecology of *T*. *cruzi* transmission in Texas and the role of animal reservoirs also need further elucidation. Among triatomine vector species caught locally, *Triatoma gerstaeckeri* is the most common, followed by *Triatoma sanguisuga*, with about two-thirds of the bugs caught noted to be PCR positive for *T*. *cruzi* [28], but *T*. *gerstaeckeri* was the species most commonly found in domestic and human habitats [29]. Dogs are being increasingly recognized as an important reservoir host for *T*. *cruzi* infection [30], but their role or importance in zoonotic transmission to humans is unknown.

Cutaneous leishmaniasis caused by *Leishmania mexicana* and transmitted by the New World sandfly vector *Lutzomyia anthophora* has also been documented in Texas but from the northeastern part of the state and the Dallas–Fort Worth metroplex [31]. A Southern Plains wood rat is a sylvatic animal reservoir in Texas [15]; however, the disease ecology is understudied, and the extent of transmission relative to Mexico (where it remains an important disease) is unclear. Recently, a cluster of cutaneous leishmaniasis caused by *Leishmania panamensis* was reported among Cuban asylum seekers in Houston who acquired infection during transit through the Panama Darien [32].

Another neglected protozoan infection found in Texas, one of great importance to women’s health and noted to be a health disparity among African American women, is trichomoniasis. In a study of tens of thousands of cervical samples of women aged 12 to 75 years, it was found that the overall detection rate was 4.5 percent [33], indicating that among an adult female population of approximately 10 million adult women [34], there would be approximately 450,000 prevalent cases in Texas. In terms of intestinal protozoan infections, DSHS estimates that there are hundreds of cases of cryptosporidiosis [35] and cyclosporiasis [36] reported each year [36] and approximately 168 cases annually of amebiasis [37]. The two most important parasitic helminth infections in Texas are cysticercosis and toxocariasis. Neurocysticercosis has been estimated to occur at a rate of 1.5–5.8 cases per 100,000 Hispanics in the US [38]. Based on a projection of almost 13 million Hispanics living in Texas by 2020 [1], it can be estimated that there will be 194–752 cases of neurocysticercosis in Texas annually, such that this disease should be considered a possible cause of epilepsy. A systematic review of toxocariasis in North America [39] failed to provide specific information on the prevalence of this disease in Texas. However, CDC surveillance studies finding 21% seroprevalence among non-Hispanic African American populations [40] would indicate that, among 3.466 million African Americans living in Texas by the year 2020 [1], there could be more than 700,000 cases. The role of seroprevalence as an indicator of active disease versus exposure in the case of *Toxocara* infection is under investigation. These are important data given the finding that toxocariasis has been linked to developmental delays and may have a role in educational achievement gaps [41].

### Arbovirus infections

Among the arboviral infections, Texas sustains the transmission of West Nile virus (WNV) infection from *Culex* mosquitoes (*C*. *quinquefasciatus* in urban areas and eastern Texas) and the transmission of dengue, chikungunya, and Zika virus infections from *Aedes* mosquitoes (especially *A*. *aegypti*).

Among the NTDs affecting Texas, WNV infection ranks at or near the top in terms of severe morbidity and mortality. Autochthonous WNV cases in Texas were first detected in 2002 after the introduction of WNV in the US (New York) in 1999 [42, 43]. During its first decade in Texas, there were over 2,000 reported cases, with a case fatality rate of 6% and an estimated economic cost to the state of $112 million [42]. A national resurgence of WNV cases in the US was observed in 2012 following a gradual decline between the years 2008 and 2011 [44]. The 2012 WNV outbreak in Texas was severe, with almost 1,900 cases (and 89 deaths), of which almost one-half occurred in the Dallas–Fort Worth metroplex [45]. Approximately 2% of the population of northern Texas was infected during the outbreak [46], with the elderly at the highest risk of neuroinvasive disease, as well as males and minority populations [45]. The economic burden of the 2012 WNV epidemic in Texas was estimated to be approximately $50 million [45]. Through genetic sequencing, it was further noted that the WNV isolates from this epidemic were not significantly different from previous strains, suggesting that external factors such as ecological or climate shifts might be responsible factors [47]. For instance, average annual temperatures during the summer of 2012 in Texas were warmer than the previous decade, with temperatures higher in Dallas than Houston [47]. It has been further noted that, during the 2000s, WNV incidence in Texas peaked in three-year cycles, possibly reflecting cyclical patterns in climate or other ecological conditions [45]. According to Texas DSHS, there were substantially fewer cases of WNV infection in 2013 and 2014 [48]. Aerial insecticide spraying was also noted to produce substantial declines in WNV disease incidence in northern Texas [49].

Beyond the acute effects of WNV infection and its association with neuroinvasive disease in Texas are its long-term effects and sequelae, investigated by Murray and her colleagues [50–55]. Patients with WNV encephalitis were found to experience hearing loss, gait and motor disturbances [50], and retinopathy [51]. In addition, WNV neuroinvasive disease was linked to long-term memory loss, fatigue, and depression [52], with fatigue linked to elevation of antiviral and proinflammatory cytokines [53]. WNV in Texas has also been shown to cause persistent infection leading to both chronic neurological and kidney disease [54], the latter associated with proteinuria and hematuria [55]. St. Louis encephalitis previously common in Texas is now associated with fewer than 10 cases annually [48]. Venezuelan equine encephalitis is also transmitted by *Culex* (as well as *Aedes*) mosquitoes, and although epizootics have struck Mexico in recent years, there are no recent human cases in Texas [15].

Texas is also at high risk of virus infections transmitted by *A*. *aegypti* and possibly *A*. *albopictus* dengue, chikungunya, and Zika virus infections. Indeed, Texas and Florida represent the two US states with the widest distribution of *A*. *aegypti* [56], while South Texas and South Florida are the only two areas of the continental US with reported year-round *A*. *aegypti* activity and disease transmission [57]. In Texas, dengue is endemic in the Rio Grande Valley, associated with limited outbreaks since 1980, including a 2005 outbreak in Brownsville (Cameron County), which represents the most southeast part of Texas on the border with Mexico [58]. But rates of infection are believed to be higher in the colonias due to the lower socioeconomic conditions there [59]. The dengue seroprevalence (immunoglobulin G [IgG]) in Cameron County, Texas, was found to be 38% among 800 randomly selected adults [60]. Outside of Brownsville, outbreaks of dengue fever due to autochthonous transmission were also found to occur in Houston, Texas, between 2003 and 2005 [61]. Overall, Texas DSHS has reported 154 dengue cases between 2003 and 2012 led by 27 cases during this period in Cameron County [62], but the reporting does not distinguish between imported versus autochthonous cases. In 2016, the Texas DSHS reported the first cases of autochthonous transmission of chikungunya and Zika virus infection. The patient with chikungunya became ill in Cameron County in November 2015, and the diagnosis was confirmed in January 2016 [63]. Texas DSHS further reported in November 2016 that Zika transmission was underway in Cameron and Hidalgo Counties, with six mosquito-transmitted cases of locally acquired Zika virus infection reported by the end of December 2016 and a locally transmitted case in December 2017, respectively [64].

### Other neglected viral and bacterial infections

Evidence for rodent hantavirus infections has been detected in both northern Mexico and South Texas [15], and sporadic cases of hantavirus pulmonary syndrome have been reported [65]. Rabies is enzootic in bats in Texas [66], and exposure to Mexican free-tailed bats is considered a risk factor for human rabies, possibly including military recruits in the San Antonio area [67]. In 2013, a man exposed to rabies in Central America was diagnosed with this condition in a US detention facility [68]. Yet another public health concern is reemerging viral infections due to declining immunization rates in Texas, such that measles and other vaccine-preventable disease outbreaks are considered possible [17]. In 2014, a case of Ebola virus infection was imported from West Africa to Dallas, Texas, followed by nosocomial transmission to two intensive care nurses, but no other infections or outbreaks followed.

With regards to neglected bacterial infections, murine typhus (typhus group rickettsiosis) caused by *Rickettsia typhi* has reemerged in Galveston (Texas Gulf Coast), where it is enzootic between fleas and opossums [69]. Previously, it was endemic in this port city due to rat populations [69]. An epidemiological analysis of typhus group rickettsiosis conducted by Murray et al. reveals that most of the cases are currently reported from South Texas, with Nueces County having the largest number of cases of the 1,762 cases between 2003 and 2013 [70]. In this study, more than one-half of the case-patients reported fleas in the home, while approximately one-third reported being bitten by a flea prior to becoming ill [70]. More recently, pediatric typhus cases have been reported in Houston [71].

Other potential neglected bacterial zoonoses that require further investigation due to their presence in animal reservoirs especially near the Mexican border include leptospirosis, bartonellosis, ehrlichiosis, and rocky mountain spotted fever [15]. Lyme borreliosis is found in Texas where it is transmitted by the *Ixodes scapularis* tick, but there is confusion between this syndrome and southern tick associated rash illness (STARI), which also causes erythema migrans but is transmitted by the lone star tick (*Amblyomma americana*) [72]. Since 2000, between 29 and 276 cases of Lyme borreliosis have been reported annually in Texas [73]. Among the neglected intestinal bacterial infections, typhoid fever cases average about 24 reported cases per year according to DSHS [74], while the number of *Vibrio* infections, either from *V*. *vulnificus* or *V*. *parahaemolyticus*, averages about several dozen infections annually [75, 76]. The last (imported) *V*. *cholera* (cholera) case was reported in 2012 [77].

According to the National Hansen's Disease (Leprosy) Registry, there were 192 reported cases of Hansen’s disease in Texas between the years 2005 and 2014, representing approximately 10% of the total cases nationally such that Texas is a leading US state in terms of numbers of leprosy cases [78]. Of interest is the finding that the armadillo found in Texas is a major zoonotic reservoir [79], although it is unclear what percentage of the Texas cases are acquired locally. Finally, syphilis is an important NTD in Texas, especially in urban areas [80, 81]. According to Texas DSHS, there were a total of 9,075 cases reported in 2016, a significant increase since 2014 and 2015 [80]. The highest rates of the infection were reported from males and African American populations [80]. In addition, there were 70 reported congenital syphilis cases in 2016 [80]. In Houston, Texas, it was recently noted that Hispanic males were affected, especially those who reported meeting partners on the internet or engaging in anonymous sex [81].

## Summary and future directions

Shown in Table 1 is a summary of the major NTDs affecting Texas. WNV infection, Chagas disease, and typhus group rickettsiosis are the most widespread vector-borne NTDs, while toxocariasis and neurocysticercosis are important zoonotic helminth infections. Trichomoniasis is a widespread parasitic protozoan and sexually transmitted NTD.

**Table 1:** Summary of the major NTDs of Texas.

Neglected Tropical Disease	Estimated Cases in Texas	Comment
Toxocariasis	>700,000 prevalent cases	Calculated based on Ref 40 CDC estimates of 21% of African Americans infected with *Toxocara* spp. multiplied by the African American population of Texas in Ref 1—the population considered at greatest risk
Trichomoniasis	Approximately 450,000 prevalent cases	Calculated using the percentage of *Trichomonas vaginalis*–infected women in Ref 33 multiplied by the at-risk population (adult females: total female population minus pediatric female population) in Ref 34
Chagas disease*	36,977 prevalent cases	Estimate from Ref 26
Syphilis	9,075 cases reported in 2016 and 70 reported congenital syphilis cases in 2016	Ref 80
WNV infection*	1,900 incident cases peaking in 2012 and annual outbreaks across Texas, resulting in 183 cases and 379 cases reported in 2013 and 2014, respectively	Refs 45 and 48
Intestinal protozoan infections: cryptosporidiosis, cyclosporiasis, amebiasis*	Approximately 1,000 cases reported annually	Refs 35–37
Neurocysticercosis*	194–752 prevalent cases	Calculated using the infection rates in Ref 38 multiplied by the estimated Hispanic population in Ref 1
Hansen’s disease (leprosy)*	192 cases reported between 2005 and 2014	Ref 78
Murine typhus*	1,762 cases between 2003 and 2013, with highest number in Nueces County (South Texas)	Refs 69–71
Lyme borreliosis*	29–276 cases annually between 2000 and 2016	Refs 72, 73
Vibrio infections*	57 cases reported in 2015	Refs 75, 76
St. Louis encephalitis*	<10 cases reported annually	Ref 48
Ebola virus infection*	3 cases in 2014	
Dengue virus infection*	Outbreaks reported in Cameron and Harris Counties	Refs 58–62
Zika virus infection*	Outbreak reported in Cameron County (South Texas) in 2016	Ref 64
Chikungunya virus infection*	Autochthonous case reported in Cameron County (South Texas) in 2015	Ref 63
Cutaneous leishmaniasis*	Sporadic cases	Ref 31
Hantavirus pulmonary syndrome*	Sporadic cases	Ref 65

* Indicates a “Texas Notifiable Condition” (https://www.dshs.texas.gov/idcu/investigation/conditions/).

Abbreviations: CDC, Centers for Disease Control and Prevention; NTD, neglected tropical disease; WNV, West Nile virus.

Overall, there is a dearth of information on the true prevalence and incidence of NTDs in Texas. A major reason for this is that information is based on reports to local health departments and the Texas DSHS, whereas most of the NTDs in Texas are seldom diagnosed or diagnosed accurately. Based on reported cases versus estimates from the CDC and other agencies, for example, less than 1% of Chagas disease cases are diagnosed in Texas, an experience similar to that reported in Mexico or elsewhere [82]. Alternatively, many of the vector-borne viral NTDs present with nonspecific symptoms or, in the case of Zika virus infection, without symptoms. The information contained here about each of the NTDs is constrained by the unevenness of the available information. Therefore, there is an urgent need to expand active surveillance activities for all of these diseases. Recently, new legislation was passed in Texas (House Bill 2055) to mandate such active surveillance activities for NTDs, the first legislation of its kind in any state.

**Fig 3:**
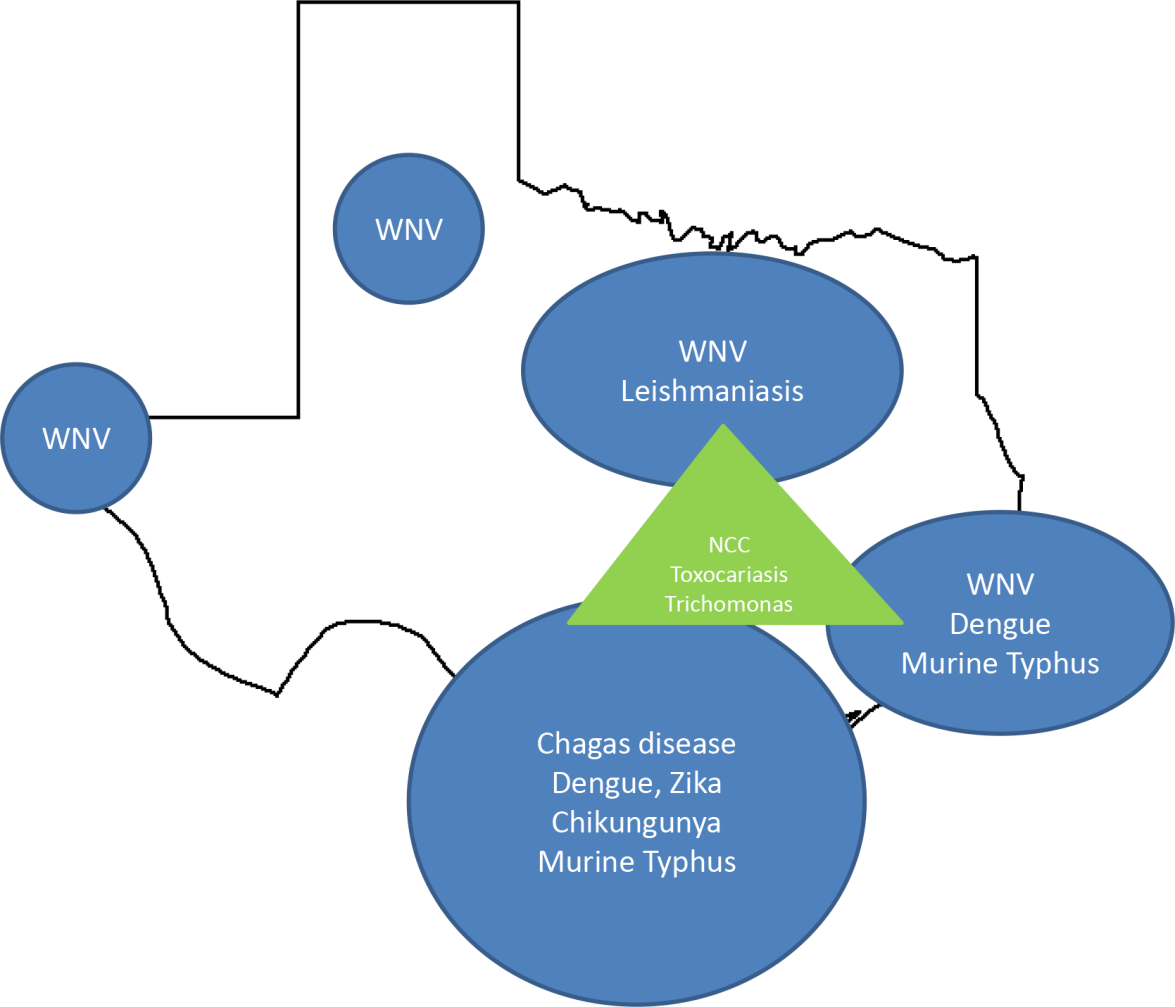
Approximate geographic distribution of the major Texas NTDs. The major diseases include the vector-borne NTDs (blue) located in South Texas, Gulf Coastal Texas, North Central Texas, the Panhandle, and West Texas, as well as non–vector-borne NTDs (green) that concentrate in the three urban areas that constitute the “Texas triangle,” i.e., Dallas, Houston, and San Antonio. NCC, neurocysticercosis; WNV, West Nile virus.

Shown in Fig 3 is a cartoon representing the approximate geographic distribution of the NTDs in Texas.

Briefly, among the vector-borne NTDs, the greatest diversity is found in southeastern Texas and includes the major arbovirus infections in addition to Chagas disease and typhus group rickettsiosis (murine typhus). In contrast, WNV infections are also widespread in the northern, central, and extreme western parts of the state. Neurocysticercosis, toxocariasis, and trichomoniasis tend to focus in poor urban areas of the more populated “Texas triangle,” which includes Dallas, Houston, and San Antonio.

As active surveillance activities expand in Texas, we can expect an expansion of data in order to make informed decisions regarding disease control and prevention. In parallel, there will be an urgency to develop improved and point-of-care diagnostics for most of the NTDs affecting Texas, in addition to better drugs, vaccines, and access to those tools [83]. Community development to improve housing, sanitation, and access to healthcare is also vitally important.

We’re still at the early stages of understanding the full extent of NTDs and emerging infectious diseases in Texas, but the trends outlined above in terms of poverty, human and animal migrations (including birds, rodents, rats, and dogs), importation of animal products, urbanization, and climate change indicate that the prevalence and incidence of NTDs could increase in the coming years.

**Key learning points**

- Texas is one of the fastest growing US states beset by the new forces of unprecedented urbanization and human migrations; poverty, inequality, and blue marble health; transborder and seaport traffic; and climate change.
- Major neglected parasitic infections include two parasitic zoonoses—cysticercosis and toxocariasis—and vector-borne diseases with autochthonous transmission of Chagas disease and cutaneous leishmaniasis.
- *A*. *aegypti*–transmitted arbovirus infection—dengue, chikungunya, and Zika have emerged in South Texas, but so far WNV infection (transmitted by Culex mosquitoes) is the most important arbovirus infection in terms of disease burden leading to chronic neurologic and renal sequelae.
- Zoonotic bacterial and viral infections are of importance, especially typhus group rickettsiosis, as is Hansen’s disease (leprosy).
- Major geographic areas of concern include South and southeast Texas but also foci in North and West Texas. There is an urgent need for active surveillance for neglected and potentially emerging diseases in these regions.

**Top five papers**

1. Garcia MN, Aguilar D, Gorchakov R, Rossmann SN, Montgomery SP, Rivera H, Woc-Colburn L, Hotez PJ, Murray KO (2015) Case report: evidence of autochthonous Chagas disease in Southeastern Texas. Am J Trop Med Hyg 92(2): 325–30.
2. Stemmer SM, Adelson ME, Trama JP, Dorak MT, Mordechai E (2012) Detection rates of trichomonas vaginalis, in different age groups, using real-time polymerase chain reaction. J Low Genit Tract Dis 16(4): 352–7.
3. Serpa JA, White AC (2012) Neurocysticercosis in the United States. Pathogens Global Health 106(5): 256–60.
4. Mann BR, McMullen AR, Swetnam DM, Salvato V, Reyna M, Guzman H, Bueno R Jr, Dennett JA, Tesh RB, Barrett DT (2013) Continued evolution of West Nile virus, Houston, Texas, USA, 2002–2012. Emerg Infect Dis 19(9): 1418–27.
5. Murray KO, Evert N, Mayes B, Fonken E, Erickson T, Garcia MN, Sidwa T (2017) Typhus Group Rickettsiosis, Texas, USA, 2003-2013. Emerg Infect Dis 23(4):645–648.
